# Probiotic *Lacticaseibacillus rhamnosus* GR-1 and *Limosilactobacillus reuteri* RC-14 as an Adjunctive Treatment for Bacterial Vaginosis Do Not Increase the Cure Rate in a Chinese Cohort: A Prospective, Parallel‐Group, Randomized, Controlled Study

**DOI:** 10.3389/fcimb.2021.669901

**Published:** 2021-07-06

**Authors:** Yongke Zhang, Jinli Lyu, Lan Ge, Liting Huang, Zhuobing Peng, Yiheng Liang, Xiaowei Zhang, Shangrong Fan

**Affiliations:** ^1^ Department of Obstetrics and Gynaecology, Peking University Shenzhen Hospital, Shenzhen, China; ^2^ Institute of Obstetrics and Gynecology, Shenzhen PKU-HKUST Medical Center, Shenzhen, China; ^3^ Shenzhen Key Laboratory on Technology for Early Diagnosis of Major Gynaecological Disease, Shenzhen, China; ^4^ College of Life Sciences, University of Chinese Academy of Sciences, Beijing, China; ^5^ BGI-Shenzhen, Shenzhen, China

**Keywords:** bacterial vaginosis, probiotics, Chinese cohort, microbiota, 16S rRNA sequencing

## Abstract

The purpose of this study was to evaluate the effectiveness of metronidazole and oral probiotics adjunct to metronidazole in the treatment of bacterial vaginosis (BV). One hundred and twenty-six Chinese women with BV were enrolled in this parallel, controlled trial, and were randomly assigned into two study arms: the metronidazole group, which was prescribed metronidazole vaginal suppositories for 7 days, and the adjunctive probiotic group, which received *Lacticaseibacillus rhamnosus* GR-1 and *Limosilactobacillus reuteri* RC-14 orally for 30 days as an adjunct to metronidazole. Clinical symptoms and Nugent scores at the initial visit, 30 days and 90 days were compared. There was no significant difference of the 30-day total cure rate between the adjunctive probiotic group (57.69%) and the metronidazole group (59.57%), with an odds ratio (OR) of 0.97 (95% confidence interval (CI), 0.70 to 1.35, *p*-value = 0.04), or of the 90-day total cure rate (36.54% vs. 48.94%, OR, 0.75; 95% CI, 0.47 to 1.19; *p-*value = 0.213). Also, no significant difference of the vaginal and faecal microbial diversity and structure between the two groups at 0, 30 or 90 days were shown based on 16S rRNA sequences. The probiotic species were rarely detected in either the vaginal microbiota or the faecal microbiota after administration which may revealed the cause of noneffective of oral probiotics. No serious adverse effects were reported in the trial. The study indicated that oral probiotic adjunctive treatment did not increase the cure rate of Chinese BV patients compared to metronidazole.

## Introduction

Bacterial vaginosis (BV), characterized by vaginal microbiota dysbiosis, is one of the most common gynaecological diseases encountered in the clinic (Kenyon et al., 2013). BV presents a particularly high morbidity rate and recurrence rate, and it affects up to 50% of women of reproductive age worldwide (Allsworth et al., 2008). BV also increases the risks of adverse pregnancy outcomes, such as preterm birth and other reproductive health sequelae (Eschenbach et al., 1988; Hillier et al., 1996; Nelson et al., 2014; Haggerty et al., 2016), and is regarded as a prevalent cause of sexually transmitted infections (STIs), including human immune-deficiency virus (HIV) infection (Martin et al., 1999; Cohen et al., 2012; Gosmann et al., 2017; Karim et al., 2019). Usually, symptoms such as thin, whitish, homogeneous and malodorous vaginal discharges are seen in BV patients in the clinic (Amsel et al., 1983), while approximately 90% of BV cases can be asymptomatic (Mlisana et al., 2012). Although approximately 60–80% of patients recover after a course of oral or vaginal metronidazole or clindamycin, the recurrence rate can reach 60% within 3 months (Bradshaw et al., 2006). Because of the high incidence of recurrence, a radical treatment method for BV is urgently required.

With the development of molecular and sequencing technologies, we have learned more about the vaginal environment, which is colonized by a community of microorganisms. Many studies have already indicated that *Lactobacillus* species dominate the vaginal microbiota of healthy women (Vásquez et al., 2002; Ravel et al., 2011; Consortium, 2012). In 2011, Ravel et al. clustered the vaginal microbial community into five groups by studying a healthy Caucasian female cohort, among which four groups were dominated by *Lactobacillus*, including *Lactobacillus crispatus*, *Lactobacillus gasseri*, *Lactobacillus jensenii* and *Lactobacillus iners* (Ravel et al., 2011). *Lactobacillus* is regarded as a protector in the vaginal environment by producing metabolites to inhibit pathogens, such as D/L-lactate, H_2_O_2_ and bacteriocins, and releasing lactic acid to acidify the vaginal environment by fermenting glycogen and maintaining a low vaginal pH (pH ≤ 4.5) to protect against pathogen invasion (Klebanoff and Coombs, 1991; Hearps et al., 2017; Tachedjian et al., 2017). Lactic acid, as one of the main metabolites of *Lactobacillus*, maintains the low pH of the vagina. Studies shown that lactic acid is significantly positively correlated with the number of *Lactobacilli* in vaginal secretions and low content in the vagina microbiota of BV patients, indicated its effect in inhibiting the growth of anaerobic bacteria (Tachedjian et al., 2017). At the same time, the ratio of the different isomers of lactic acid, D/L-lactic acid, also affects upper reproductive tract infections by regulating the matrix metalloproteinase MMP-8 (Witkin et al., 2013; Tozetto-Mendoza et al., 2020). Hydrogen peroxide (H_2_O_2_), another metabolite of *Lactobacillus*, may also play an important role in maintaining the vaginal anaerobic environment and inhibiting the invasion of pathogenic microorganisms (Al-Mushrif and Jones, 1998; Basu Thakur et al., 2019). As a result, the lack of *Lactobacillus* will reduce the defence mechanism of vagina. When the abundance of *Lactobacillus* species is strikingly reduced and replaced by a greatly increased proportion of anaerobic microorganisms, such as *Gardnerella vaginalis*, *Prevotella bivia*, *Atopobium vaginae* and *Mycoplasma hominis*, this dysbiosis will likely develop further as BV (Verhelst et al., 2004; Ravel et al., 2011). Thus, the vaginal microbiota turns out to be closely associated with the pathophysiological process of BV.

Probiotic supplementation has recently emerged as a novel biotherapy targeted to the vaginal microbiota (Cohen et al., 2020). Several clinical studies have shown that lactobacilli-containing probiotics are effective adjuncts to antibiotics in treating BV and other infection symptoms, whether administered vaginally or orally (Recine et al., 2016; Bohbot et al., 2018; Cianci et al., 2018; Russo et al., 2018; Cohen et al., 2020; Wijgert et al., 2020). The oral *Lactobacillus* strains could survival in the gastrointestinal tract and be eliminated from the body with stool, then small amount of *Lactobacillus* remains at the perianal area could transmission and colonized into the vagina, where it can reproduce in larger quantities (Reid et al., 2001b; Bohbot and Cardot, 2012; Yefet et al., 2020). Moreover, studies also found that even if it is not colonized in the vagina, oral probiotics also have a positive impact on the vaginal microbiota *via* promote self-recovery of *Lactobacilli* in the vagina through immune regulation or altering the internal environment of the human body (Eslami et al., 2016; Ho et al., 2016).

*L. rhamnosus* GR-1 and *L. reuteri* RC-14 were isolated and developed by Reid et al. for vaginal health (Reid et al., 1987) and have come into China as a formulation of dietary supplements in recent years. The efficacy of these two probiotic strains has been evaluated in Brazilian, Black African, and Canadian patients, among others (Anukam et al., 2006; Martinez et al., 2009; Bisanz et al., 2014; Macklaim et al., 2015), but Asian data are lacking. Since previous studies have shown that the clinical efficacy of probiotic intervention varied in different races, the question of whether a probiotic strain extracted from European women can function equally in Chinese women still needs further research.

A prospective, parallel‐group, randomized, controlled study was designed here and aimed to evaluate the effect of oral administration of *L. rhamnosus* GR-1 and *L. reuteri* RC-14 after a 7-day course of metronidazole in the treatment of BV. BV patients treated with traditional antibiotic therapy served as controls. Vaginal and faecal microbiota were identified using the 16S rRNA gene sequencing approach to explore the mechanisms of action.

## Materials and Methods

### Study Population

This study was a single-centre, prospective, parallel-group, randomized, and controlled study. Women who came to the gynaecological clinic of Peking University Shenzhen Hospital, China, between March 2019 and June 2019 with abnormal vaginal discharge symptoms were recruited, and eligible subjects were informed of the study protocol and enrolled in the study. The inclusion criteria were women aged between 18 and 65 years old, premenopausal women, women with a history of sexual activity, and women with a Nugent’s Gram stain score of 7 or higher. The exclusion criteria were mixed vaginitis, such as vulvovaginal candidiasis (VVC), *Trichomonas vaginalis* (TV) infection, *Chlamydia trachomatis* (CT) infection or gonococcal vaginitis; planning for or being pregnant; breast-feeding; pelvic inflammatory disease; allergy to metronidazole; currently using antibiotics; long-term use of contraceptives or immunosuppressants or anaphylactic constitution; and a history of systemic organic diseases or psychiatric diseases. The study was conducted in accordance with the Declaration of Helsinki, was approved by the Medical Ethnic Committee of PKUSZH (with the Unique Protocol ID: PUshenzhenH2018-016) and was first posted on ClinicalTrials.gov (NCT03894813) on March 26, 2019. Informed consent was obtained from all subjects involved in the study.

The sample size was calculated based on the non-inferiority test (https://www.cnstat.org/samplesize/12/), which referred to Gregor Reid et al. (Reid et al., 2001a; Reid et al., 2003b; Reid et al., 2003a), who carried out studies with the same *Lactobacillus* strains administered orally to women with BV. The relevant parameters were determined as follows: test level α = 0.05, test power 1-β = 0.80, Pt = 0.90, Pc = 0.75, Nt : Nc = 1 : 1, boundary value Δ = -0.0.068 (excellent), and Fisher’s exact test. As a result, the sample size of each group was calculated as 50 patients, 100 patients in total. Considering a loss to follow-up rate of 20%, a sample size of 120 patients was finally determined, with 60 patients in each group.

This study was a single-centre randomized controlled trial with two arms allocated at a ratio of 1 : 1. SPSS 13.0 software was applied to generate a random number table, and the random order was assigned depending on the order of enrolment.

### Study Design

After obtaining written informed consent, Nugent scoring was carried out as a diagnostic code for BV, and those with Nugent scores ≥ 7 were informed of the study protocol and enrolled in the study. The participants were first asked to complete a questionnaire on demographic characteristics, reproductive health and sexual behaviour. They were subsequently randomly assigned to either the metronidazole group or the adjunctive probiotic group according to the random number table. The study was not blinded to the researchers or the patients. Starting from the enrolment day, the participants in the adjunctive probiotic group received orally administered probiotic drinks containing *L. rhamnosus* GR-1 and *L. reuteri* RC-14 (≥1 × 10^9^ CFU per day, for 30 days) and vaginally administered metronidazole suppositories (0.2 g per day, for 7 days), and the participants in the metronidazole group received metronidazole vaginal suppositories only. The *L. rhamnosus* GR-1 and *L. reuteri* RC-14 in the BV therapeutic are not sensitive to metronidazole. Clinical follow-up visits were scheduled at 30 and 90 days after starting treatment. The intervention products were dispensed by the investigator at the initial visit, and the compliance of participants was assessed by counting returned containers and filling out a questionnaire. The feasibility, acceptability and adherence to the administered products; vaginal symptoms; and adverse events (AEs) were also assessed by self-report in medication diaries and questionnaires at follow-up visits. Therapy was initiated after the enrolment visit and suspended when menstruation began and continued immediately when it finished. Patients were asked to avoid sexual intercourse and vaginal douching during the first 7 days of treatment with metronidazole suppositories.

### Clinical Evaluation

General information, including age, employment status, smoking history, relationship status, childbirth history, sexual activity, disease history, and allergy history, of all enrolled participants was evaluated before treatment. Clinical signs and symptoms of BV, including homogeneous and thin vaginal discharge, unpleasant odour (such as a “fishy” smell) and vulvar discomfort (such as itching or burning), were recorded at 0 day, 30 days and 90 days. The efficacy outcome was evaluated by the cure rate according to per-protocol (PP) analysis, which was the percentage of participants who did not present with BV (defined by Nugent score < 7) at any of the follow-up visits. Those with a Nugent score ≥ 7 in the 30-day follow-up was excluded from the trial, but when we evaluate the overall recurrence rate of the 90-day follow-up, these participants were still included in. At each follow-up visit, patients were requested to report any unexpected symptoms during the study period. AEs were recorded in the case report form (**Table S1**).

The diagnosis criteria of BV were based on Nugent Gram stain scoring of vaginal smears (Nugent et al., 1991). The Gram-stained smear slides were examined microscopically, and the number of bacterial cells with different morphotypes was counted under 1000 × magnification. Each slide was identified according to Nugent score. A Nugent score of 0-6 was considered BV negative and of 7-10 was considered BV positive. The Gram stains were analysed in a double-blind manner by two experienced cytology technicians. The pH value of vaginal discharge was measured on site using a pH paper strip (pH range 3.8-5.4; Shanghai SSS Reagent Co., Ltd., Shanghai, China).

### Vaginal and Faecal Microbiota Sample Processing and Analysis

At enrolment and each follow-up time point, two vaginal samples were collected by specimen collection swabs (iClean, Huachenyang (Shenzhen) Technology Co., Ltd., Guangdong, China): one for the screening of BV by Nugent scoring and to measure vaginal pH, and the other was stored in 3 ml of buffer (MGI (Shenzhen) Technology Co., Ltd, Guangdong, China) at -80°C and later used for vaginal microbiota DNA profiling. All the patients were asked to self-collect a sample of faeces at enrolment and each follow-up visit using a self-collection bag containing the transport medium from BGI. The faecal samples were also stored at -80°C immediately upon receipt at the laboratory.

Total DNA of vaginal and faecal samples was extracted using a QIAamp DNA Mini Kit (Qiagen, Hilden, Germany) and DNA concertation was determined by a Qubit Fluorometer, and the purity and integrity of the gDNA were measured by 0.6% agarose gel electrophoresis. The DNA samples were stored at -80°C for further usage. Qualified genomic DNA samples were configured with the specific primers 8F (AGAGTTTGATCCTGGCTCAG) and 518R (TTACCGCGGCTGCTGGCAC), and the variable regions V1 to V3 of the 16S rRNA gene were amplified by PCR. Subsequently, Agencourt AMPure XP magnetic beads were used to purify PCR amplicons, which were dissolved in TE buffer (pH = 8.0) and labelled to complete library construction. An Agilent 2100 Bioanalyzer was used to detect the insert fragment size, and then the products were sequenced on the HiSeq2500 platform (Illumina, USA) using the PE300 module.

High-quality data were obtained after filtration, and tags spliced by overlapping reads were clustered into OTUs using PARSE with a 97% cut-off. Then, the representative OTU sequences extracted by USEARCH Global were annotated to the Greengenes database (V201305) by RDP Classifier (v2.2). To obtain higher resolution of the species profile, we performed BLAST on the representative OTU sequences with the cut-off of 98% identity and 95% coverage. The species with highest identity and coverage were selected. If two or more species with same identity and coverage were annotated, we would go to details of the sequence, and the identified species sequences from high-qualified whole genome of ATCC isolates would be selected priority. The abundances of the bacterial taxonomy at the genus and species levels were then calculated. R (v3.2.1) was applied to perform the alpha diversity analysis, which was expressed by the Shannon-Wiener index. Unweighted UniFrac principal coordinate analysis (PCoA) was performed by QIIME (v1.80). The hierarchical clustering of the vaginal and faecal microbiota of BV patients before treatment was analysed by the complete linkage method and R package ‘pheatmap’.

### Statistical Analysis

Statistical analysis was performed to calculate the difference between groups. The nonparametric Wilcoxon-Test was used for two independent group comparisons, and the Kruskal test was used for multiple group comparisons. *P*-values were generated to represent significance (*p*-value < 0.01).

## Results

### Participant Flow and Recruitment Baseline Data

**Figure 1:**
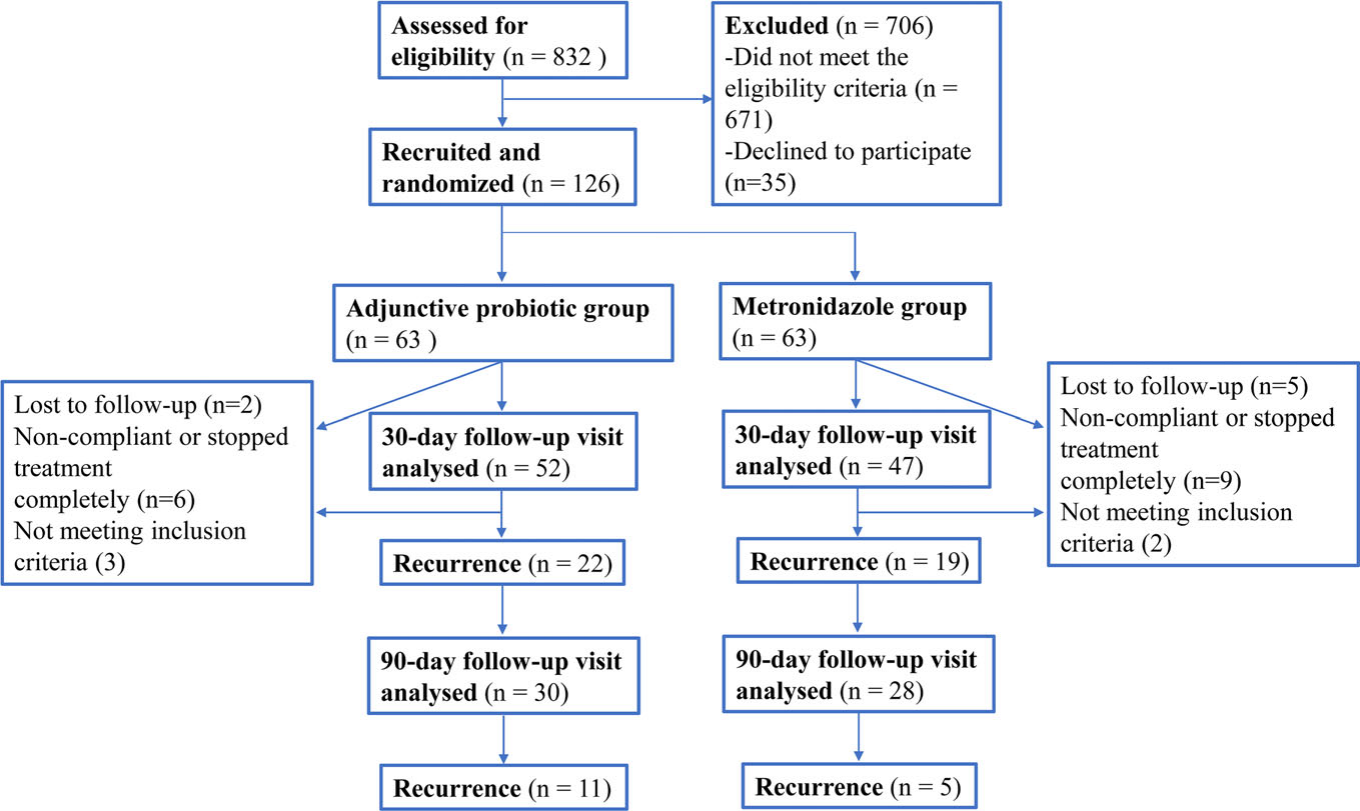
Flow chart showing numbers of participants at each stage of the trial.

In total, 832 patients were eligible for BV screening. After diagnosis based on the Nugent score, 706 patients were excluded, and 126 women diagnosed with BV (Nugent score 7-10) were enrolled for randomization in this trial (**Figure 1**). Twenty-seven patients dropped out of the study, among whom three were found to be pregnant during the experiment (one was diagnosed at the 30-day visit and the other two were diagnosed at the 90-day visit), two patients required antibiotic therapy, and twenty-two refused to return to the hospital for the follow-up visits (16 at the 30-day visit and 6 at the 90-day visit). As a result, 99 patients participated in the 30-day visit, and 58 participated in the 90-day visit. The differences in participant characteristics at baseline between the two groups enrolled in this study were evaluated to eliminate individual bias (**Table 1**). Basic information, including age, employment status and smoking history, was not significantly different between the two groups (*p*-value > 0.05), nor was the relationship status, birth history, current sexual activity or condom use. Additionally, clinical symptoms and laboratory parameters were also compared between the two groups. No significant difference in external genital itching and burning, vaginal pH value and Nugent score was observed between the metronidazole group and adjunctive probiotic group. Moreover, the composition of the vaginal and faecal microbiota of the two groups at baseline was also taken into consideration to control the quality of the trial (**Tables S2**, **S3**). For the vaginal microbiota, no significant difference in the relative abundance of any genera was observed. For the faecal microbiota, although the *p*-value of the genus *Parabacteroides* was below 0.05, the false detection rate (FDR) was 0.051, which indicated that there was no significant difference. No significant difference in the abundances of the other genera in the faecal microbiota at baseline was observed. Therefore, no individual bias was involved in this study at the enrolment stage.

**Table 1:** Demographic and clinical characteristics at baseline in the study population.

Clinical characteristics	Value		*P* value
	Adjunctive probiotic group (n=52)	Metronidazole group (n=47)	
Age (years, mean ± SD)	34.2 ± 7.0	33.3 ± 7.5	0.543
Employment status, n (%)			
Stable job	43 (82.69)	41 (87.23)	0.5296
Unemployed or unstable work	9 (17.31)	6 (12.77)	
Smoking history, n (%)	5 (9.62)	2 (4.26)	0.3
Current relationship status, n (%)			
Married	36 (69.23)	26 (55.32)	0.1536
Single, divorced or others	16 (30.77)	21 (44.68)	
Childbirth history, n (%)			
At least one previous birth	31 (59.62)	26 (55.32)	0.666
Never give birth before	21 (40.38)	21 (44.68)	
Sexual activity, n (%)			
Regularly sexual activity (vaginal sex) which is at least 4 times per one month	28 (53.85)	20 (42.55)	0.262
Not had sexual activity in the last half of the year or vaginal sex less than 4 times per one month	24 (46.15)	27 (57.45)	
Condom using, n (%)	24 (46.15)	18 (38.30)	0.4295
Clinical symptoms or signs, n (%)			
Thin, whitish, homogeneous vaginal discharge	33 (63.46)	30 (63.83)	0.97
Fishy smell or other bad Vaginal odour	41 (78.85)	40 (85.11)	0.4186
Itching	29 (55.77)	20 (42.55)	0.1894
Burning	5 (9.62)	7 (14.89)	0.4228
Laboratory test, n (%)			
Vaginal pH (mean ± SD)	5.05 ± 0.29	5.03 ± 0.30	0.7902
Nugent score (mean ± SD)	7.8 ± 0.87	8.0 ± 0.87	0.2907

### Efficiency by Nugent Score Evaluation

**Figure 2:**
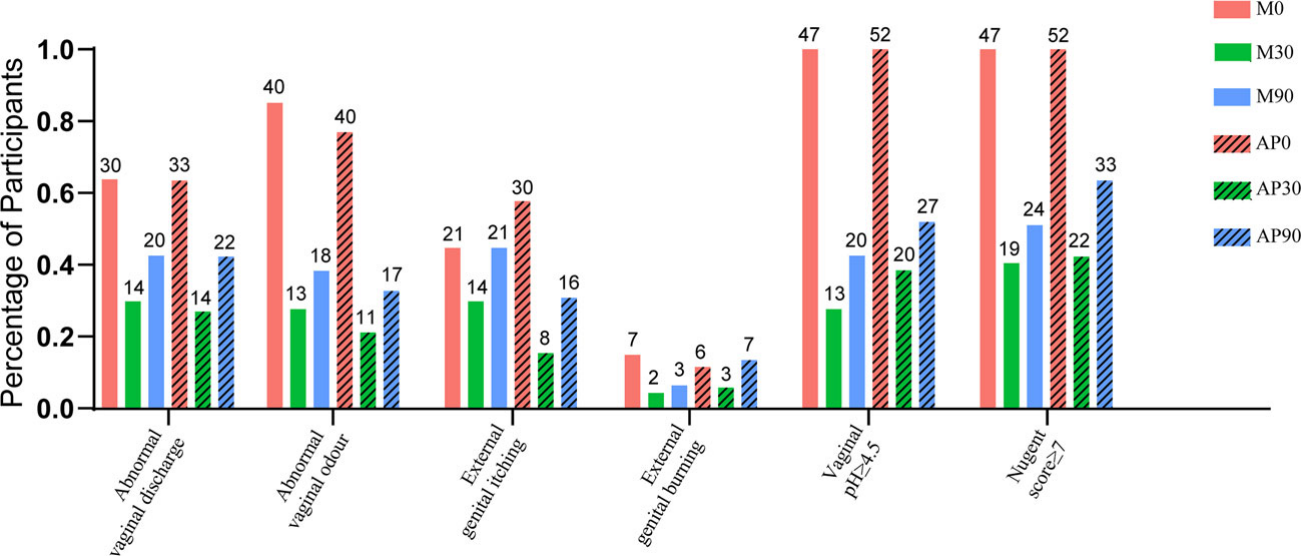
The percentage of the participants with detected BV-associated symptoms (abnormal vaginal discharge, abnormal vaginal odour, external genital itching and external genital burning) and laboratory parameters (vaginal pH ≥ 4.5 and Nugent score ≥ 7) at different time-point of the trial were shown. The number marked on the top of the bar denote the case number.

The percentage of participants with detected BV-associated symptoms at different time points of the trial is shown in **Figure 2**. The symptoms of BV, including abnormal vaginal discharge, abnormal vaginal odour, external genital itching and external genital burning, were improved in both the adjunctive probiotic group and the metronidazole group, and laboratory parameters such as vaginal pH ≥ 4.5 and Nugent score ≥ 7 were also shown in fewer participants after 30 days of intervention. The total cure rates of the adjunctive probiotic group and the metronidazole group at 30 days were 57.69% and 59.57%, respectively; however, there was no significant difference in the 30-day total cure rate, with an OR of 0.97 (95% CI, 0.70 to 1.35 and *p*-value = 0.04), or in the 90-day total cure rate (36.54% vs. 48.94%, OR, 0.75; 95% CI, 0.47 to 1.19; *p*-value = 0.213) between groups.

### Vaginal Microbiota

**Figure 3:**
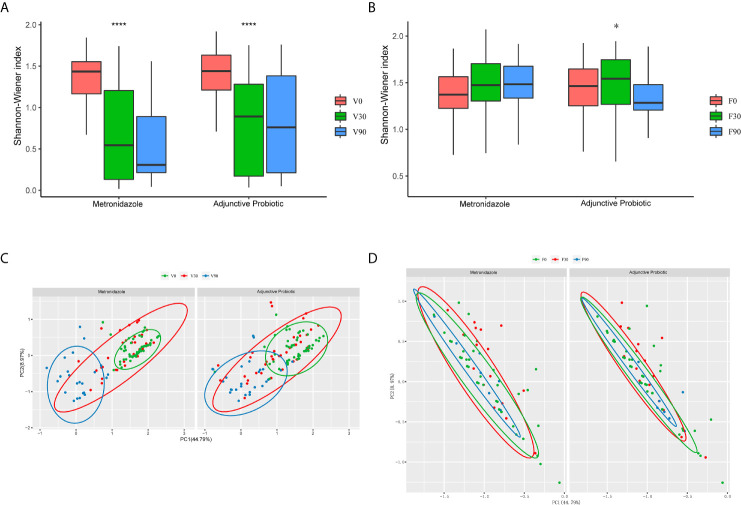
The microbial diversity of the vaginal and fecal samples. The α-diversity of the vaginal **(A)** and fecal **(B)** microbiota presented by the box-plot of Shannon-Winner index. Wilcoxon test was used to do the statistical analysis of each two independent groups and Krustal test for multiple groups. The asterisks indicated the difference of multi-group. They denote the significant difference among all the three bars below. *stands for *p*-value < 0.05; ****stands for *p*-value < 0.0001. Principal coordinate analysis (PCoA) of the vaginal **(C)** and fecal **(D)** samples was also performed. Each dot represents one individual sample, and the green ones stands for those collected at baseline, while red for 30 days and blue for 90 days.

Based on the 16S rRNA sequences, vaginal and faecal microbiota were analysed. After obtaining the OTUs, the α-diversity was calculated and is shown in **Figure 3**. The results indicated a significant decrease in α-diversity in the vaginal microbiota (**Figure 3A**) after 30 days of treatment in both the metronidazole group and the adjunctive probiotic group, and for the sample collected at 90 days after treatment, a continuous decrease in α-diversity was observed in the metronidazole group but not in the adjunctive probiotic group. No significant difference in α-diversity was noticed between the two groups at 0 day (*p*-value = 0.75), 30 days (*p*-value = 0.2) or 90 days (*p*-value = 0.11). The results indicated that the vaginal microbiota was affected by metronidazole treatment, which was administered to all participants, but oral probiotics likely had no influence on microbial diversity. For the faecal microbiota (**Figure 3B**), the α-diversity showed no significant difference in the metronidazole group during the whole trial but increased significantly at 30 days in the adjunctive probiotic group. This observation suggested that oral *Lactobacillus* could affect the gut microbiota but not the vaginal microbiota. In addition, PCoA was applied to investigate the correlation and differentiation between the two groups (**Figures 3C, D**). Vaginal samples at 0 day and 90 days were gathered and separated from each other and mixed with the samples at 30 days, which means that the microbiota structure at 30 days is a transition stage and is altered after 90 days. Coincident with diversity, there was no difference in the faecal microbiota between the two groups.

**Figure 4:**
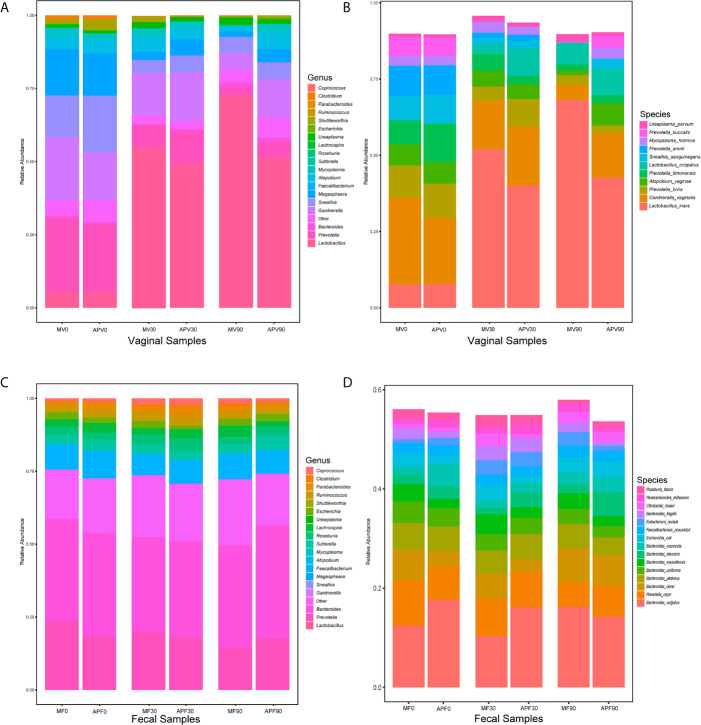
The microbial taxonomy of vaginal and fecal microbiota at genus and species level. Based on the 16S rRNA sequences, the vaginal **(A)** and fecal **(C)** bacterial genus with average relative abundance above 0.01, and bacterial species abundance above 0.01 detected from vaginal **(B)** and fecal **(D)** microbiota were also shown. MV0, MV30 and MV90 represent the vaginal samples collected at 0-day, 30-day and 90-day in the metronidazole group, respectively. MF0, MF30 and MF90 represent the fecal samples collected at 0-day, 30-day and 90-day in the metronidazole group, respectively. APV0, APV30 and APV90 represent the vaginal samples collected at 0-day, 30-day and 90-day in the adjunctive probiotic group, respectively. APF0, APF30 and APF90 represent the fecal samples collected at 0-day, 30-day and 90-day in the adjunctive probiotic group, respectively.

The composition of the microbial community was also identified in all samples (**Figure 4**). The dominant bacterial genera in the vaginal microbiota at baseline were all BV-associated bacteria, such as *Prevotella*, *Bacteroides*, *Gardnerella*, *Sneathia* and *Mycoplasma*, while *Lactobacillus* were dominant after 30 days in both the metronidazole group and the adjunctive probiotic group (**Figure 4A**). However, the decreasing trend in BV-associated bacteria and the increasing trend in *Lactobacillus* were not maintained for 90 days in the adjunctive probiotic group. At the species level. *L. iners* was the most abundant *Lactobacillus* species in the community, which inferred that it could be the key species contributing to the recovery of BV (**Figure 4B**). No significant alteration was shown in faecal microbial structure in the two groups during the trial (**Figures 4C, D**).

### Evaluation of the Key Species

**Figure 5:**
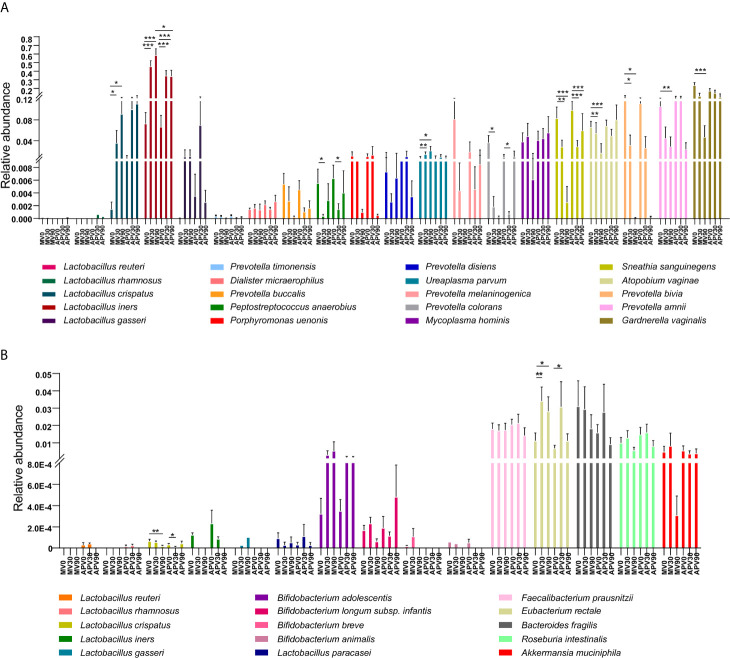
The abundance of the top abundant bacterial species and key *Lactobacillus* species in vaginal microbiota. The relative abundances of bacterial species with abundance above 0.01 in the vaginal environment of BV patients have been evaluated **(A)**. The relative abundance of vaginal dominant *Lactobacillus* species **(B)**, including *L. crispatus, L. gasseri* and *L. iners*, as well as the intervention probiotic species (*L. rhamnosus* and *L. reuteri*) in the vaginal microbiota **(B)** have also been explained. Wilcoxon test was used to do the statistical analysis of each two independent groups, and *p*-value was noted by stars above the bars. *stands for *p*-value *< 0.05*; **stands for *p*-value < 0.01; ***stands for *p*-value < 0.001.

To better understand the variation in the microbiota, the differences in the species with relative abundances above 0.01% were further analysed (**Figure 5A**). *G. vaginalis*, *Prevotella bivia*, *Prevotella amnii*, and *Atopobium vaginae* were significantly reduced after treatment in the metronidazole group, while decreased abundance was also shown in the adjunctive probiotic group but without significance. *Sneathia sanguinegens* was inhibited effectively in both groups. However, *Mycoplasma hominis* and *Ureaplasma* species were not efficiently suppressed after treatment, indicating that treatment with metronidazole and a probiotic adjunct to metronidazole may be invalid. The relative abundance of vaginal dominant *Lactobacillus* species, including *L. crispatus*, *L. gasseri* and *L. iners*, was also evaluated in the study (**Figure 5A**). Another dominant species, *L. jensenii*, was not shown here since we did not detect it in the current cohort. A significant increase in *L. iners* was shown in the two groups, while *L. crispatus* increased significantly only in the adjunctive probiotic group. However, probiotic species (*L. rhamnosus* and *L. reuteri*) were rarely detected in both vaginal and faecal microbiota. Some other probiotic lactobacilli species, as well as butyric-producing bacterial species, showed no significant difference after intervention in the faecal microbiota, with the exception of the significant increase in *Eubacterium rectale* and decrease in *L. crispatus* (**Figure 5B**). However, these results could not be caused by the probiotic intervention since the same results were observed in the metronidazole group.

### The Association of Intervention Outcome With the Microbiota Present Before Treatment

**Figure 6:**
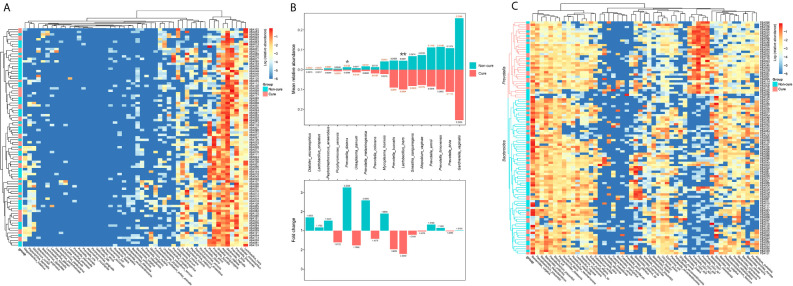
The hierarchical cluster of the vaginal and fecal microbiota of the participates at 0-day. The heatmap of the hierarchical cluster analysis of the bacterial species with relative abundance above 0.001 in vaginal **(A)** and fecal **(C)** microbiota of each participants. The colour was corresponded with the colour bar at right side defined by log of the relative abundance. The left bar was represented the cure (red) and non-cure (blue) outcome of the participants. The relative abundance of the bacterial species above 0.01 in vaginal microbiota in cure group and non-cure group, and their multiple relationship were also calculated **(B)**. The number marked at the end of the bar in the upper plot was the mean relative abundance, and the number in the lower plot was the fold change. Wilcoxon test was used to do the statistical analysis of each two independent groups, and p-value was noted by stars above the bars. *stands for *p*-value < 0.05; **stands for *p*-value < 0.01.

To further investigate the reason for the inefficiency of the treatment, we performed hierarchical clustering of the vaginal and faecal microbiota of the participants at baseline when they were all diagnosed with BV. Basically, we grouped them into a cure group and a non-cure group and included the top 50 most abundant bacterial species in the analysis. No clear clusters were found in either the vaginal microbiota or the faecal microbiota on the basis of cure and non-cure outcomes (**Figures 6A, C** and **Figure S1**). For the vaginal microbiota, the relative abundance of *L. iners* was two-fold more abundant and significantly higher in the cure group than in the non-cure group, which indicated that *L. iners* was a potential microbial marker for the efficacy of metronidazole for the treatment of BV. On the other hand, *Prevotella disiens* presented significantly higher abundance in the non-cure group, demonstrating that its presence may prevent BV cure by the current treatment method; the same may be true for *Mycoplasma hominis*, which was almost two-fold more abundant in the non-cure group. Additionally, species belonging to *Prevotella* exhibited high variations in their contribution to the cure of BV (**Figure 6B**). Moreover, we also analysed some important pathogens that appeared frequently in the vaginal microbiota of BV patients, leading to or associated with other gynaecological complications, which were hard to identify through microscopy. Their occurrence ratio in the participants was calculated (**Table S4**). Interestingly, we found that *G. vaginalis* was present in all samples, although with different abundances. This finding suggested that metronidazole and probiotic adjunctive therapy suppressed *G. vaginalis* rather than eliminated it, and this may be a key reason for recurrence. Two *Mycoplasma* species were detected in BV patients, including *Mycoplasma hominis* and *Mycoplasma genitalium*. The occurrence ratio of *M. hominis* was decreased after treatment in both groups. Although *M. genitalium* was shown in only two BV patients, it was not cleared after treatment. This indicated that this *Mycoplasma* species may not be sensitive to the current therapy. Moreover, no reduction was found in the occurrence ratio of *Ureaplasma* species after treatment (56.37% vs 59.29% on average), nor in their relative abundance (**Figure 5A**). *Atopobium*, another key BV-associated bacteria, was also examined in this study. Although the abundance of *A. vaginae* decreased in the metronidazole group after treatment, it was still 86.45% on average among the participants after treatment. Additionally, *Escherichia. coli* was also detected in 34.72% of our vaginal samples, and the occurrence ratio did not decrease at either 30 days or 90 days, which demonstrated that the patients had a potential risk of catching aerobic vaginitis. For the faecal microbiota, all the participants were clearly grouped into two clusters: one dominated by Bacteroides and the other dominated by *Prevotella* (**Figure 6C**). This result was consistent with previous studies that found that two enterotypes existed in the human gut microbiota. However, the clusters were not associated with the treatment outcomes, indicating that the enterotype had no effect on the current therapy.

### Safety

No serious AEs were recorded over the course of the study. According to the case report form, 11 participants reported vaginal itching or burning, all of whom were diagnosed with vulvovaginal candidiasis and were subsequently treated with antifungal drugs. Considering anti-fungal drug is mainly targeted on fungal but not bacteria, and the microbiota we discussed in this study is focusing on bacteria, these cased still counted as the follow-up cases. However, the whole bacterial community can be interreacted with fungal community and influenced each other, whether antifungal drugs influenced the faecal microbiota and vaginal microbiota directly still need further studies. In addition, 6 participants reported mild vaginal bleeding, 3 exhibited abnormal vaginal discharge, and 2 reported mild lower abdominal pain. In addition, haematochezia, opsomenorrhea, abdominal distention and frequent urination were found in one patient each.

## Discussion

In general, our study applied two treatments, metronidazole and a probiotic treatment as an adjunct to metronidazole, for BV patients. No significant difference was presented between the metronidazole group and the probiotic adjunctive group in the total cure rate either at 30 days (59.57% vs 57.69%) or at 90 days (48.94% vs 36.54%), indicating an ineffective outcome of oral administration of the probiotics used in this study. In addition, the effect of oral administration of these two lactobacilli for 30 days on the vaginal and faecal microbiota of Chinese women was also nonsignificant. However, this clinical trial still verified that the oral administration of *L. rhamnosus* GR-1 and *L. reuteri* RC-14 daily adjuncts to metronidazole for BV treatment is safe for Chinese BV patients, and no serious AEs appeared.

Adjunctive probiotic therapy for BV treatment has emerged in recent years, and some clinical trials using probiotics containing *L. rhamnosus* GR-1 and *L. reuteri* RC-14 have been carried out, but the outcomes varied. In 2006, 125 Black African women diagnosed with BV were treated with oral metronidazole and randomized to receive oral GR-1 and RC-14 or placebo. Of all subjects who returned for the 30-day follow-up, 88% were cured in the antibiotic/probiotic group, compared to 40% in the antibiotic/placebo group (*p* < 0.001) (Anukam et al., 2006). Another clinical trial also carried out by Gregor Reid et al. based on Nugent score in a 2009 study recruited 64 participants (32 randomized in the probiotic group and 32 in the control group) and indicated that more BV cure rate [80% vs 50% (p = 0.001)] and better microbiota (75% vs 34.4%, p = 0.011) were shown in the probiotics group at the end of treatment than the control group after treatment with a single dose of tinidazole (2 g) supplementation adjunct to 2 daily probiotic (*L. rhamnosus* GR-1 and *L. reuteri* RC-14) capsules when compared with those in the placebo group (*p* < 0.01) (Martinez et al., 2009). Moreover, oral probiotics could lower the α -diversity index of the vaginal microbiota and increase the abundance of *L. crispatus* and *L. iners* (Macklaim et al., 2015). Although these outcomes of trials were all different from ours, the increase in *L. iners* was clear, as shown in the current study. Consistent with our study, the rates of BV did not differ between the probiotic and placebo groups after oral administration of *L. rhamnosus* GR-1 and *L. reuteri* RC-14 to pregnant women. Additionally, there were no differences in the α-diversity or the composition of the vaginal microbiota between or within the probiotic and placebo groups at different time points (Husain et al., 2020), nor in the cytokine and chemokine levels, as shown in a very recent similar study (Yang et al., 2020).

Although previous studies indicated that oral probiotics could contribute to the recovery of the vaginal microbiota and that probiotic species or strains could be detected in the vagina after oral probiotic administration (Anukam et al., 2006; Vujic et al., 2013; Heczko et al., 2015; Macklaim et al., 2015; Russo et al., 2018), contrary views still exist (Happel et al., 2020; Husain et al., 2020). A recent study from Reid et al. demonstrated that oral intake of *L. rhamnosus* GR-1 and *L. reuteri* RC-14 did not change the vaginal microbiota significantly in low-risk pregnant women through the assessment of both Nugent Gram stain scoring and high-throughput sequencing (Yang et al., 2020). Although *L. rhamnosus* was detected in the vagina of 98% of the women at 13 weeks of gestation, its abundance did not change with probiotic treatment, and no *L. reuteri* was found. Moreover, another clinical trial on pregnant women from the UK also showed no differences in the proportion of women colonized with probiotic strains, nor in the α-diversity or composition of the vaginal microbiota, between probiotic adjunctive and metronidazole groups (Husain et al., 2020). In the current study, we also determined the relative abundance of *L. rhamnosus* and *L. reuteri* in vaginal and faecal samples. After 30 days of probiotic intervention, *L. rhamnosus* was detected in only one vaginal sample, as well as *L. reuteri*. In healthy Chinese women, it is rare to find *L. rhamnosus* and *L. reuteri* in their vaginal microbiota, and this was true in the BV patients in this study. Therefore, it is possible that these two probiotic strains cannot survive in Chinese women’s vaginal environment. As a result, this probiotic treatment was not as effective as presented in European or Black African women (Jespers et al., 2015; Tomusiak et al., 2015). Moreover, unfortunately, among faecal samples, *L. rhamnosus* and *L. reuteri* showed very limited abundance in only 3% and 5% of the participants, which could be very difficult to influence the gut microbiota and vaginal microbiota. Therefore, probiotic failure to appear in the gut and vagina may be the conclusive reason that this probiotic intervention was ineffective in Chinese BV patients.

The vaginal microbiota of healthy women has been grouped into 5 community state types (CSTs), among which four were dominated by *Lactobacillus* species, including *L. crispatus*, *L. gasseri*, *L. iners* and *L. jensenii* (Ravel et al., 2011). A previous study demonstrated that native dominant *Lactobacillus* species could persistently colonize the vagina, while others (Vallor et al., 2001), such as the *L. rhamnosus* and *L. reuteri* investigated in this study, are more likely to be transient. Hence, more potential *Lactobacillus* strains belonging to dominant species should be developed for women’s vaginal health. For instance, *L. crispatus* CTV-05 (Lactin-V) is an investigational new drug (IND)-proven probiotic strain applied to prevent the recurrence of BV. The phase IIb trial results indicated that the use of Lactin-V after vaginal metronidazole treatment could significantly lower the incidence of BV recurrence when compared to placebo (Cohen et al., 2020).

The dose of lactobacilli was another possible reason for the lack of significant results (Huang et al., 2014). The total numbers of *L. rhamnosus* GR-1 and *L. reuteri* RC-14 in the probiotic product in this study were examined and were 8 × 10^9^ CFU/sachet/day at the beginning of the trial and declined to 1 × 10^9^ CFU/sachet/day at the end of the trial. The decrease in the number of lactobacilli could play a role in the effectiveness of the product. Furthermore, the timing of probiotic use, sample collection and subsequent visits, as well as dietary habits and daily routines, could also alter the results of the trial (Dausset et al., 2018; Marcotte et al., 2019). Moreover, the antifungal medication prescribed to 11 participants who reported vaginal itching or burning and were diagnosed with vulvovaginal candidiasis in the trial, could have some effect on faecal and vaginal microbiome, and this may also indicate the limitation of the study.

## Conclusions

The study indicated that daily oral use of *L. rhamnosus* GR-1 and *L. reuteri* RC-14 as adjuncts to metronidazole vaginal suppositories did not increase the BV cure rate in Chinese patients compared to treatment with metronidazole alone, which may result from the lack of therapeutic probiotic species in the vaginal and faecal microbiota after administration.

## Data Availability Statement

The datasets presented in this study can be found in online repositories. The names of the repository/repositories and accession number(s) can be found below: [https://www.ncbi.nlm.nih.gov/PRJNA730591].

## Ethics Statement

The studies involving human participants were reviewed and approved by the Medical Ethnic Committee of PKUSZH. The patients/participants provided their written informed consent to participate in this study.

## Author Contributions

SF and XZ designed and conducted the research. YZ, LH and LG carried out the clinical trail. XZ, YZ and JL analyzed the data and wrote the paper. ZP, and YL provided critical revisions of the article for intellectual content. All authors contributed to the article and approved the submitted version.

## Funding

This trial was funded by the Shenzhen Healthcare Research Project (grant number SZXJ2018080) and the Shenzhen Science and Technology Innovation Commission (grant number JCYJ20190809101409603 and JCYJ20200109140614667). The funder of the study had no role in the study design, data collection, data analysis, data interpretation or writing of the report.

## Conflict of Interest

Author LG was employed by company BGI-Shenzhen.

The remaining authors declare that the research was conducted in the absence of any commercial or financial relationships that could be construed as a potential conflict of interest.
