# Supplementary material for: Probiotic Lacticaseibacillus rhamnosus GR-1 and Limosilactobacillus reuteri RC-14 as an Adjunctive Treatment for Bacterial Vaginosis Do Not Increase the Cure Rate in a Chinese Cohort: A Prospective, Parallel‐Group, Randomized, Controlled Study
Source: Front Cell Infect Microbiol. 2021 Jul 6;11:669901. doi: 10.3389/fcimb.2021.669901 (PMC8291149; doi:10.3389/fcimb.2021.669901)
Supplement: Supplementary file 1 [file DataSheet_1.zip › Supplementary Materials/Supplementary files captions.docx]

# Supplementary Material

Table S1: The case report forms of adverse events that occurred during the trial.

Table S2: Wilcoxon test results of the vaginal microbiota at the species level between the metronidazole group and the adjunctive probiotic group at 0 days.

Table S3: The Wilcoxon test results of the faecal microbiota at the species level between the metronidazole group and the adjunctive probiotic group at 0 days.

Table S4: The number of detected cases and occurrence ratio (denoted in parentheses) of important pathogens that appeared frequently in the vaginal microbiota of BV patients, leading to or associated with other gynaecological complications, in metronidazole and adjunctive probiotic groups at 0 days, 30 days and 90 days.

Figure S1: The hierarchical cluster of the vaginal (A) and faecal (B) microbiota of the participants at baseline ranked by cure and non-cure outcomes.
