# Supplementary figures and images for: Probiotic Lacticaseibacillus rhamnosus GR-1 and Limosilactobacillus reuteri RC-14 as an Adjunctive Treatment for Bacterial Vaginosis Do Not Increase the Cure Rate in a Chinese Cohort: A Prospective, Parallel‐Group, Randomized, Controlled Study
Source: Front Cell Infect Microbiol. 2021 Jul 6;11:669901. doi: 10.3389/fcimb.2021.669901 (PMC8291149; doi:10.3389/fcimb.2021.669901)

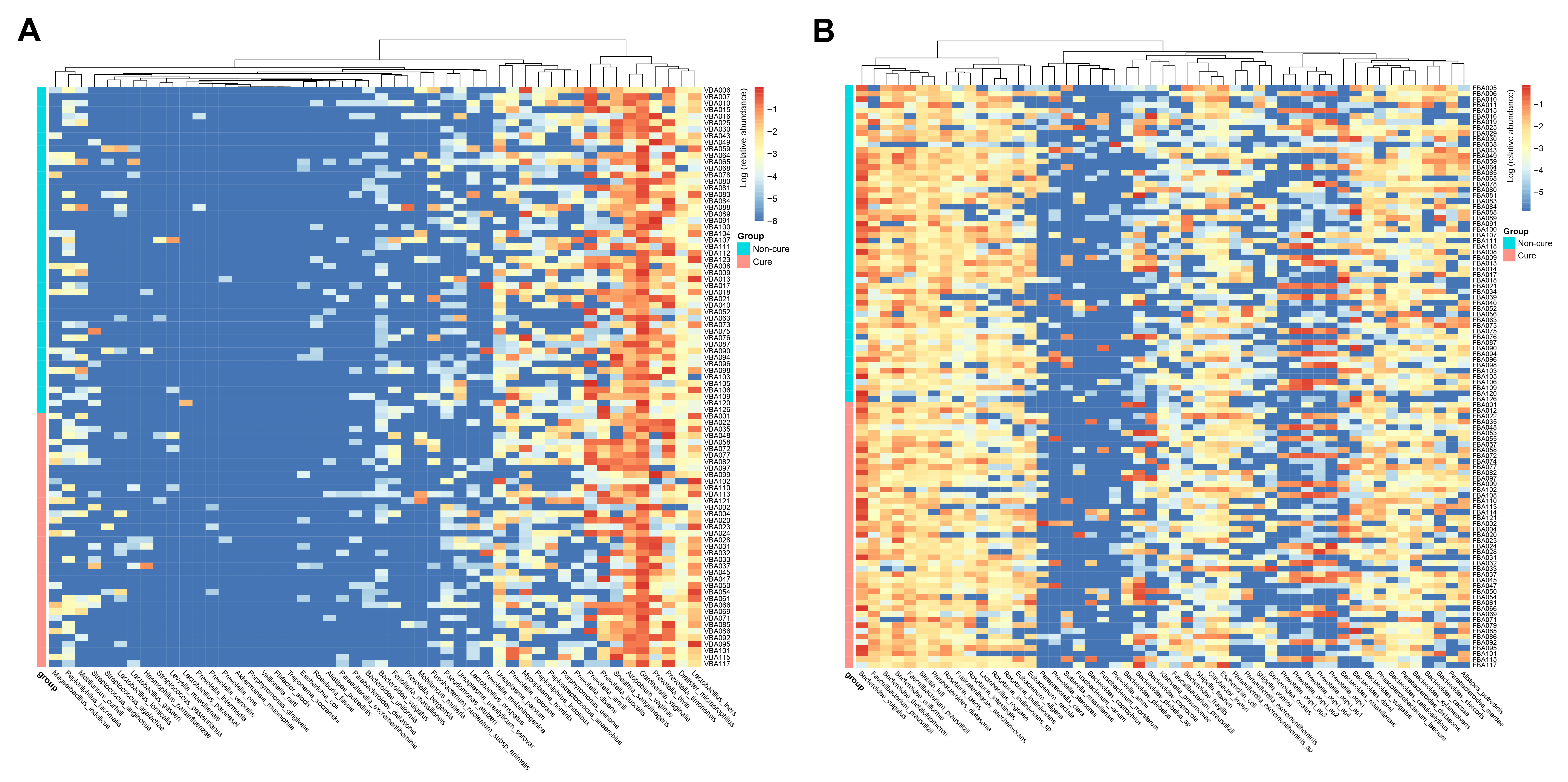

Supplement: Supplementary file 1 [file DataSheet_1.zip › Supplementary Materials/Supplementary_figure_1.jpg]
